# Reference ventricular dimensions and function parameters by cardiovascular magnetic resonance in highly trained Caucasian athletes

**DOI:** 10.1186/s12968-023-00910-7

**Published:** 2023-02-09

**Authors:** Alicia M. Maceira, Jose V. Monmeneu, M. Pilar López, M. Pilar García, Laura Higueras, M. Dolores Masiá, Araceli Boraita

**Affiliations:** 1 Cardiovascular Imaging Unit, Cardiology Department, Ascires Grupo Biomédico, Valencia, Spain; 2 Cardiology Department, Hospital San Juan, Alicante, Spain; 3 Cardiology Department, Spanish Sports Health Protection Agency, Madrid, Spain

**Keywords:** Athletes, Cardiac magnetic resonance, Left ventricle, Right ventricle, Reference values

## Abstract

**Background:**

Data regarding cardiovascular magnetic resonance (CMR) reference values in athletes have not been well determined yet. Using CMR normal reference values derived from the general population may be misleading in athletes and may have clinical implications.

**Aims:**

To determine reference ventricular dimensions and function parameters and ratios by CMR in high performance athletes.

**Methods:**

Elite athletes and age‐ and gender‐matched sedentary healthy controls were included. Anatomical and functional variables, including biventricular volumes, mass, systolic function, wall thickness, sphericity index and longitudinal function were determined by CMR.

**Results:**

A total of 148 athletes (29.2 ± 9.1 years; 64.8% men) and 124 controls (32.1 ± 10.5 years; 67.7% men) were included. Left ventricular (LV) mass excluding papillary muscles was 67 ± 13 g/m^2^ in the control group and increased from 65 ± 14 g/m^2^ in the low intensity sport category to 83 ± 16 g/m^2^ in the high cardiovascular demand sport category; P < 0.001. Regarding right ventricular (RV) mass, the data were 20 ± 5, 31 ± 6, and 38 ± 8 g/m^2^, respectively; P < 0.001. LV and RV volumes, and wall thickness were higher in athletes than in the control group, and also increased with sport category. However, LV and RV ejection fractions were similar in both groups. LV and RV dimensions, wall thickness and LV/RV ratios reference parameters for athletes are provided.

**Conclusions:**

LV and RV masses, volumes, and wall thicknesses are higher in athletes than in sedentary subjects. Specific CMR reference ranges for athletes are provided and can be used as reference levels, rather than the standard upper limits used for the general population to exclude cardiomyopathy.

**Supplementary Information:**

The online version contains supplementary material available at 10.1186/s12968-023-00910-7.

## Introduction

Long-term physical training leads to a number of structural and functional cardiac adaptations usually known as ‘athlete’s heart’, a physiological process that includes biventricular dilatation and left ventricular (LV) hypertrophy to facilitate increased stroke volume in response to exercise [1]. This process has great individual variation across age, genders and sport disciplines [2]. Furthermore, there is substantial overlap between physiological adaptation and cardiomyopathy in the so-called grey area, which can render the distinction between athlete’s heart and cardiac disease difficult, especially in extreme forms of sport-related ventricular hypertrophy and dilatation, which may require differential diagnosis with hypertrophic [3], dilated [4] and arrhythmogenic [5] cardiomyopathies.

Cardiomyopathy is recognized as one of the leading causes of sudden death in athletes [6]. Consequently, regular medical evaluation is progressively being implemented in athletes, including cardiac imaging in those athletes with high suspicion of heart disease [7]. Distinction of pathological findings from physiological adaptation is relevant to minimize the risk of sudden death and because of the career changing decisions of elite athletes that can derive from it.

Cardiovascular magnetic resonance (CMR) has excellent accuracy for the measurement of biventricular volumes, mass and function, stress induced perfusion defects, and for analysis of coronary origin. It also has a unique capability of detecting myocardial fibrosis. CMR provides a comprehensive evaluation of the heart and is an appropriate technique for the preparticipation assessment of athletes in whom symptoms or signs suggest heart disease, and of asymptomatic athletes with either abnormal examination, abnormal electrocardiogram (ECG), or definite (or high suspicion for) family history of inheritable heart disease [8].

The use of CMR normal reference values derived from the general population may be misleading in highly trained athletes. Reliable CMR reference values obtained in these athletes are thus mandatory to reduce inconclusive reports caused by the grey area between physiological adaptation and cardiopathy, to prevent athletes being barred from sports because of false positive findings and to prevent reassurance of athletes in whom cardiac pathology goes undetected.

The aims of this study were, firstly, to assess differences between high-performance athletes and age and gender-matched controls with respect to variables including LV and right ventricular (RV) morphological and functional parameters, secondly, to detect significant predictors of ventricular dimensions and function in athletes and, finally, to establish reference values for biventricular parameters normalized for independent influences such as age, gender, height, body surface area and type of sport in athletes.

## Methods

### Subjects included

This was an observational and prospective study carried out with CMR at 1.5 T in which elite athletes in regular competition with ≥ 10 h training per week, and age‐ and gender‐matched sedentary healthy controls were included. Sport disciplines were classified according to their characteristics [9]. All subjects, males and females, were white athletes and above 18 years of age. Athletes were active on their training programs at the time of the study (typically > 2 days and < 7 days since last training session). The control group consisted of subjects exercising ≤ 3 h per week, age and gender matched, and also matched for height and weight. In this group, the majority of subjects performed activities including Nordic walking, yoga, Pilates, tennis, paddle, swimming, ballroom dances, hiking, cycling, running, etc. This is the normal physical activity for the majority of subjects in Spain. As a result, this was considered as an appropriate control group [10]. Exclusion criteria included standard contraindications for CMR, personal history of cardiovascular risk factors such as hypertension [11], diabetes mellitus [12], smoking habit, dyslipidemia [13], use of illicit substances, family history of cardiac disease, personal history of cardiac, respiratory or renal disease, presence of ECG abnormalities other than those reported as compatible with normal physiological adaptations in athletes [14] and/or evidence of cardiovascular structural or functional abnormalities on the CMR study. The study was carried out according to the principles of the Declaration of Helsinki and was approved by the institutional ethics committee. Each subject gave informed written consent.

Data collected included age, gender, sport discipline, years of training, average training hours per week, height, weight, and body surface area that was calculated according to the Mosteller formula [15]. All CMR studies were analyzed and reported by two cardiologists with at least 10-year experience in CMR and individual diagnosis was made by consensus. Only patients with normal CMR were included. Whenever consensus of absence of cardiopathy could not be reached, a third cardiologist was invited for deciding the final diagnosis, but these cases were not included in the study.

CMR was performed according to standardized CMR imaging protocols [16]. CMR was performed in 1.5 T CMR scanners (Avanto and Symphony, Siemens Healthineers, Erlangen, Germany) using front and back surface coils and retrospective ECG triggering. CMR scans were acquired by operators with at least 4-year experience in CMR.

Images were analyzed with a specific software (Medis, Leiden, The Netherlands) by two experienced observers (more than 10 yr experience). End-diastole and end-systole were selected from the time-volume curve. LV and RV endocardial and epicardial borders were manually delineated in all planes in end-diastole, while in end-systole only the respective LV and RV endocardial borders were delineated. LV papillary muscles were also delineated in end-diastole in all the planes in which they appeared.

LV mass (LVM) was calculated from the end-diastolic frames. Papillary muscle mass was also calculated, and LVM values were obtained both including and excluding papillary muscle mass. LV segmental wall thickness was quantified in diastole for all 16 segments [17]. Papillary muscles were excluded when measuring ventricular volumes. RV mass was calculated form the end-diastolic frames. RV free wall thickness was measured in the mid-lateral segment (segment 7) in all the subjects included [18]. End-diastolic and end-systolic volumes were calculated for both the LV and the RV. The LV and RV outflow-tracts were included for calculating the ventricular volumes. These parameters were indexed to body surface area, height and height^2.7^ for comparative analysis. LV ejection fraction (LVEF) and RV ejection fraction (RVEF) were calculated as (end-diastolic volume – end-systolic volume)/end-diastolic volume. LV and RV atrioventricular plane displacement were measured in the lateral wall of both the LV and RV and in the septum and expressed as a percentage of the end-diastolic length. End-diastolic and end-systolic LV and RV sphericity indexes were also calculated. Other definitions included: relative wall mass (RWM): mass relative to end-diastolic volume (g/mL); wall thickness dispersion index: the standard deviation of the wall thickness of all 16 segments; atrioventricular plane descent (AVPD): the length (apex to lateral or septal AV groove, respectively) in diastole minus this length in systole divided by the length in diastole * 100; sphericity index: LV basal radial length/longitudinal length, measured in the apical 4-chamber views during end-diastole and end-systole; anterior apical to basal ratio: the wall thickness of the anterior wall in the apical segment divided by the wall thickness in the anterior wall at the basal segment [19]. First pass myocardial perfusion at rest was visually assessed. Presence of regional wall motion abnormalities were excluded with CMR.

### Statistical analysis

Categorical variables were described by their absolute (n) and relative frequencies (%) and continuous variables by the mean and standard deviation or median and interquartile range according to normality distribution. Univariate normality assumptions were verified with the Shapiro–Wilk test. Differences between athletes and controls with respect to variables regarding baseline characteristics and ventricular dimensions and function were assessed using analysis of variance (ANOVA) or Kruskal–Wallis test for continuous variables, as appropriate, and Chi-square for categorical variables. Two-way ANOVA or Kruskal–Wallis test, as appropriate, were used to analyze differences in LV and RV dimensions and function parameters according to age, gender and intensity of sport. Simple linear regression was used to analyze variations in ventricular dimensions and function according to age, gender and sport, to model the data and to construct reference ranges as mean and 95% confidence intervals. A level of statistical significance of 0.05 was applied in all the statistical tests. The data were analyzed using the statistical package SPSS (v22.0, Statistical Package for the Social Sciences, International Business Machines, Inc., Armonk, New York, USA).

## Results

A total of 148 athletes (29.2 ± 9.1 years with age range 18–50 years; 64.8% men) and 124 controls (32.1 ± 10.5 years; 67.7% men) were included in the study. The reasons for athletes to perform a CMR included post-acute pericarditis to rule out myocardial involvement (n = 11); for minor symptoms, defined as subjective decrease in sport performance, atypical chest pain, palpitations (n = 56); for suspicion of coronary anomaly (n = 2); to rule out intracavitary mass (n = 9); for suspicion of RV anomalies in echocardiogram, such as RV dilation, wall motion abnormalities, or systolic dysfunction (n = 34); and for suspicion of LV anomalies in echocardiogram, such as LV hypertrophy, LV dilatation, LV systolic dysfunction, or hypertrabeculation (n = 36).

Athletes and controls were well balanced with regard baseline clinical characteristics. Table 1 shows anthropometric variables of controls and athletes, as well as training information of athletes and distribution across sport groups. Sport disciplines were categorized according to Pelliccia´s classification [9] in low, medium and high intensity sports. Among athletes, mean hours per week of training were 16.4 ± 7.9 and mean years of training 10.6 ± 5.3, with no differences with respect to sport category.

**Table 1:** Anthropometric variables in athletes and controls

Group	All		Males		Females		P value		
	Controls	Athletes	Controls	Athletes	Controls	Athletes	Group	Gender	
N	124	148	84	96	40	52			
Age (years)	32.1 ± 10.5	29.2 ± 9.1	32.1 ± 10.5	29.6 ± 10.1	32.2 ± 10.4	28.6 ± 8.4	NS	NS	
Weight (Kg)	74 ± 14	70 ± 12	79 ± 12	76 ± 11	63 ± 13	61 ± 10	NS	< 0.001	
Height (cm)	173 ± 9	175 ± 11	177 ± 8	180 ± 9	164 ± 7	166 ± 8	NS	< 0.001	
BSA (m^2^)	1.87 ± 0.22	1.85 ± 0.21	1.96 ± 0.18	1.94 ± 0.18	1.69 ± 0.17	1.68 ± 0.16	NS	< 0.001	
BMI (g/m^2^)	21.0 ± 3.2	20.2 ± 2.7	21.9 ± 2.7	21.0 ± 2.3	19.1 ± 3.3	18.7 ± 2.6	NS	< 0.05	
Sport classification (n, %)									
Low intensity		12 (8.1%)		7 (7.3%)		5 (9.6%)	NS		
Medium intensity		70 (47.3%)		40 (41.7%)		30 (57.7%)			
High intensity		66 (44.6%)		49 (51.0%)		17 (32.7%)			
Years of training (years)		10.6 ± 5.3		10.6 ± 6.0		10.6 ± 3.8	–	NS	
Average training (h/week)		16.4 ± 7.9		15.1 ± 6.6		18.5 ± 9.0	–	NS	

Dynamic component	Low (< 50%)			Medium (50–75%)			High (> 75%)		
Static component		Males	Females		Males	Females		Males	Females
Low (< 10%)									
	Riflery	1	1	Fencing	1		Badminton		1
	Golf	2	1	Table tennis	2	1	Field hockey		4
							Orienteering	3	1
							Running (long distance)	19	14
							Soccer	11	1
Moderate (10–20%)									
	Archery	1	1	Rugby	1		Basketball	3	3
	Equestrian		1	Running (sprint)	2		Running (middle distance)	6	5
				Ultra racing	1		Swimming	4	
							Team handball	1	
							Tennis	1	
High (> 30%)									
	Gymnastics		3	Wrestling	3	1	Boxing	2	
	Martial arts	2	2				Canoeing	1	
	Sailing	1	2				Kayaking	1	
	Weight lifting		2				Rowing	3	1
							Cycling	14	4
							Triathlon	10	3

Distribution of athletes with respect to sport classification. *BSA* body surface area, *BMI* body mass index. *Sport classification according to [9]

LV morphology parameters, as well as LV wall thickness ratios, LV/RV ratios and RWM for ventricular parameters in controls and athletes according to sport category are reported in Table 2 and Additional file 1: Table S1. No significant differences were seen for the majority of LV and RV parameters between controls and the low intensity sport group. LV mass excluding papillary muscles was 67 ± 13 g/m^2^ in control group and increased from 65 ± 14 g/m^2^ in the low intensity sport category to 83 ± 16 g/m^2^ in the high intensity sport category; P < 0.001. Similarly, LV end-diastolic volume was 77 ± 14 mL/m^2^ in the control group and increased from 79 ± 10 to 106 ± 19 mL/m^2^ according to sport category, respectively; P < 0.001 and for LV end-systolic volume, these numbers were 27 ± 9 mL/m^2^, 28 ± 7 mL/m^2^ and 40 ± 10 mL/m^2^, respectively; P < 0.001. LVEF was 66 ± 7% in the control group, without significant differences between groups. Regardless segment, wall thickness was significantly greater in athletes than in the control group, even in the low intensity group, and increased with the intensity of sport. Interestingly, apical-to-basal wall thickness ratios did not differ between controls and athletes. LV relative wall mass excluding papillary muscles was 0.83 ± 0.14 g/mL in the control group and decreased from 0.81 ± 0.15 to 0.78 ± 0.18 g/mL, respectively, according to the intensity of sport; P < 0.05.

**Table 2:** Left ventricular morphology and function parameters, wall thickness dispersion index, relative wall mass and left ventricular/right ventricular ratios in controls and athletes classified with respect to sport category

Sport category according to intensity					
	Control	Low intensity	Medium intensity	High intensity	P
LV EDV (mL)	140 ± 31 ^‡§^	140 ± 23 ^‡§^	184 ± 38 ^*†§^	196 ± 46 ^*†‡^	< 0.001
LV EDV/BSA (mL/m^2^)	77 ± 14 ^‡§^	79 ± 10 ^‡§^	103 ± 16 ^*†^	106 ± 19 ^*†^	< 0.001
LV ESV (mL)	50 ± 19 ^‡§^	51 ± 13 ^‡§^	66 ± 18 ^*†§^	77 ± 24 ^*†‡^	< 0.001
LV ESV/BSA (mL/m^2^)	27 ± 9 ^‡§^	28 ± 7 ^‡§^	37 ± 9 ^*†^	40 ± 10 ^*†^	< 0.001
LV EF (%)	66 ± 7	65 ± 7	64 ± 6	63 ± 6	NS
LVM excluding PM (g)	118 ± 33 ^‡§^	118 ± 35 ^‡§^	145 ± 40 ^*†§^	163 ± 41 ^*†‡^	< 0.001
LVM excluding PM/BSA (g/m^2^)	67 ± 13 ^‡§^	65 ± 14 ^‡§^	80 ± 19 ^*†^	83 ± 16 ^*†^	< 0.001
LV RWM excluding PM (g/mL)	0.83 ± 0.14 ^‡§^	0.81 ± 0.15	0.78 ± 0.15 ^*^	0.78 ± 0.18 ^*^	< 0.05
LV Max WT (mm)	8.4 ± 1.32 ^‡§^	9.2 ± 2.2	9.4 ± 1.8 ^*^	9.8 ± 1.7 ^*^	< 0.001
WTDI (mm)	0.89 ± 0.23 ^‡§^	1.01 ± 0.42	1.03 ± 0.38 ^*^	1.12 ± 0.38 ^*^	< 0.01
WT AB ratio	0.78 ± 0.05	0.79 ± 0.07	0.80 ± 0.06	0.79 ± 0.08	NS
PM mass, total (g)	4.7 ± 1.34 ^‡§^	4.8 ± 1.3 ^§^	5.4 ± 1.6 ^*§^	6.2 ± 1.6 ^*†^	< 0.001
PM mass/BSA (g/m^2^)	2.5 ± 0.65 ^‡§^	2.8 ± 0.68	3.1 ± 0.80 ^*^	3.2 ± 0.76 ^*^	< 0.001
LV AVPD, septal (%)	20 ± 4	18 ± 4	20 ± 3	20 ± 4	NS
LV AVPD, lateral (%)	18 ± 4	18 ± 3	19 ± 4	18 ± 4	NS
LV sphericity index, ES	2.44 ± 0.38	2.41 ± 0.23	2.33 ± 0.30	2.35 ± 0.37	NS
LV sphericity index, ED	2.01 ± 0.21	2.07 ± 0.18	2.00 ± 0.19	1.97 ± 0.21	NS
LV / RV EDV ratio	1.00 ± 0.12	0.99 ± 0.10	1.00 ± 0.10	1.02 ± 0.11	NS
LV / RV ESV ratio	0.92 ± 0.23	0.92 ± 0.15	0.94 ± 0.18	0.98 ± 0.20	NS
LV / RV EF ratio	1.07 ± 0.10	1.05 ± 0.08	1.05 ± 0.09	1.04 ± 0.08	NS

*LV* left ventricle, *EDV* end-diastolic volume, *BSA* body surface area, *ESV* end-systolic volume, *EF* ejection fraction, *LVM*: left ventricular mass, *PM* papillary muscles, *RWM* relative wall mass, *Max WT* maximum wall thickness, *WTDI* wall thickness dispersion index, *WT AB ratio* global apical to basal wall thickness ratio, *PM mass*, total papillary muscle mass, *AVPD* atrioventricular plane displacement, *ES* end-systole, *ED* end-diastole, *RV* right ventricle

Post-Hoc analysis: *significant differences with “Control” group; ^†^significant differences with “Low intensity” sport group; ^‡^significant differences with “Medium intensity” sport group; §significant differences with “High intensity” sport group

With regard to RV (Table 3 and Additional file 1: Table S2), RV mass was 20 ± 5 g/m^2^ in the control group and increased from 31 ± 6 g/m^2^ in the low intensity sport to 38 ± 8 g/m^2^ in the high sport category; P < 0.001. Similarly, RV end-diastolic volume was 78 ± 15 mL/m^2^ in the control group and increased from 81 ± 12 to 105 ± 17 mL/m^2^ according to sport category, respectively; P < 0.001 and for RV end-systolic volume, these numbers were 30 ± 9 mL/m^2^, 31 ± 9 mL/m^2^ and 41 ± 11 mL/m^2^, respectively; P < 0.001. RVEF was 62 ± 7% in the control group, without significant differences between groups. RV wall thickness was higher in athletes than in the control group, regardless intensity of sport; P < 0.001.

**Table 3:** Right ventricular morphology and function parameters in controls and athletes classified with respect to sport category

Sport category according to intensity					
	Control	Low intensity	Medium intensity	High intensity	P
RV EDV (mL)	147 ± 35 ^‡§^	145 ± 30 ^‡§^	184 ± 40 ^*†§^	203 ± 42 ^*†§^	< 0.001
RV EDV/BSA (mL/m^2^)	78 ± 15 ^‡§^	81 ± 12 ^‡§^	104 ± 19 ^*†^	105 ± 17 ^*†^	< 0.001
RV ESV (mL)	56 ± 19 ^‡§^	56 ± 15 ^‡§^	72 ± 18 ^*†^	81 ± 24 ^*†^	< 0.001
RV ESV/BSA (mL/m^2^)	30 ± 9 ^‡§^	31 ± 9 ^‡§^	41 ± 10 ^*†^	41 ± 11 ^*†^	< 0.001
RV EF (%)	62 ± 7	61 ± 6	61 ± 6	61 ± 7	NS
RVM (g)	39 ± 10 ^†‡§^	54 ± 11 ^*§^	62 ± 17 ^*§^	72 ± 18 ^*†‡^	< 0.001
RVM/BSA (g/m^2^)	20 ± 5 ^†‡§^	31 ± 6 ^*‡§^	36 ± 8 ^*†^	38 ± 8 ^*†^	< 0.001
RV RWM	0.30 ± 0.05 ^†‡§^	0.35 ± 0.06 ^*^	0.35 ± 0.07 ^*^	0.36 ± 0.08 ^*^	< 0.001
RV WT (mm)	3 ± 1 ^‡§^	4 ± 0.8	4 ± 0.7^*^	4 ± 0.7^*^	< 0.001
RV AVPD, lateral (%)	27 ± 6	24 ± 6	24 ± 5	25 ± 6	NS
RV sphericity index, ES	2.35 ± 0.49	2.44 ± 0.43	2.45 ± 0.55	2.29 ± 0.52	NS
RV sphericity index, ED	2.34 ± 0.52	2.34 ± 0.33	2.46 ± 0.47	2.41 ± 0.43	NS

*RV* right ventricle, *EDV* end-diastolic volume, *BSA* body surface area, *ESV* end-systolic volume, *EF* ejection fraction, *RVM* right ventricular mass, *RWM* relative wall mass, *RV WT* RV free wall thickness (measured in segment 7), *AVPD* atrioventricular plane displacement, *ES* end-systole, *ED* end-diastole

Post-Hoc analysis: *significant differences with “Control” group; ^†^significant differences with “Low intensity” sport group; ^‡^significant differences with “Medium intensity” sport group; §significant differences with “High intensity” sport group

**Fig. 1:**
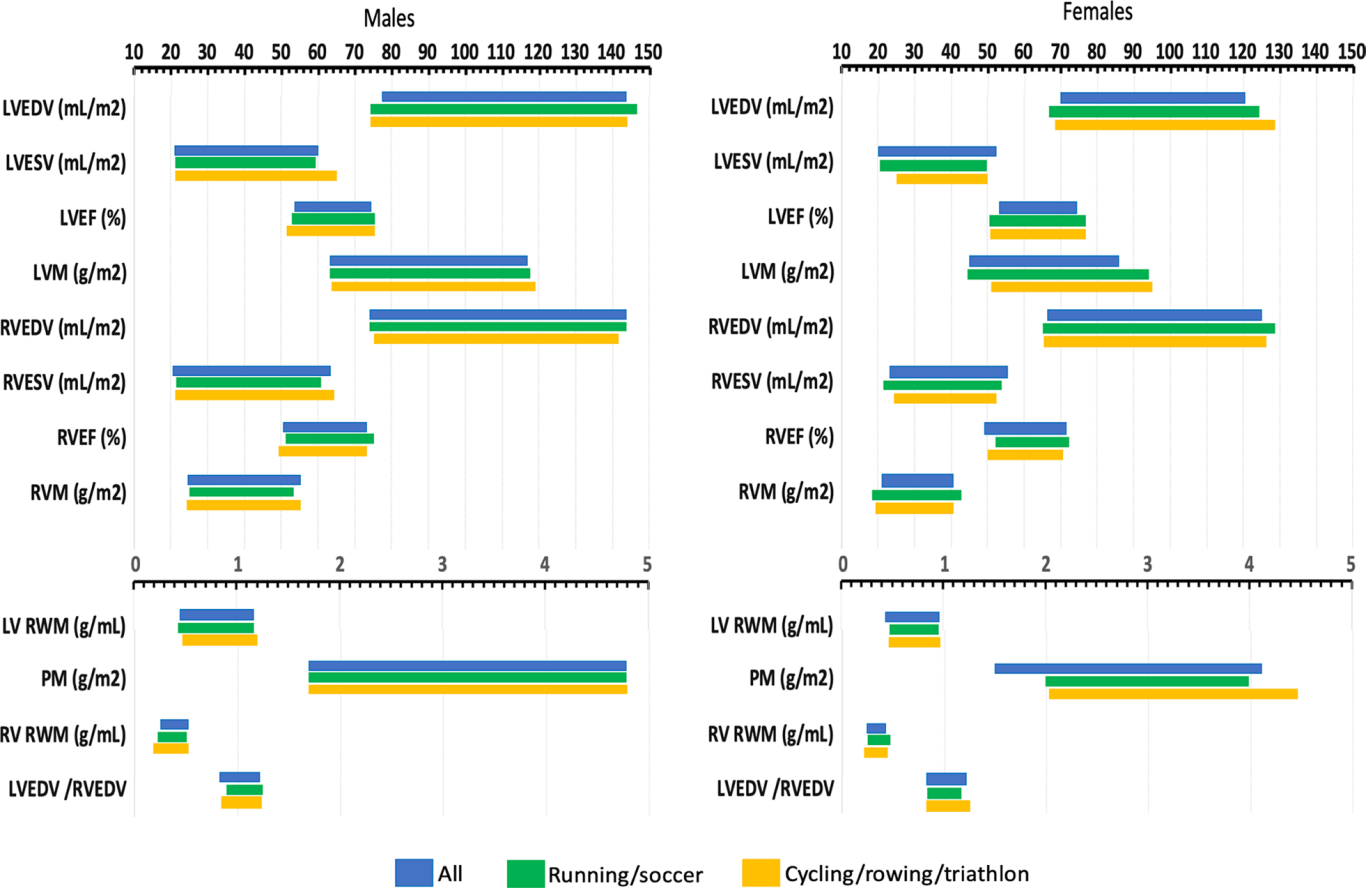
Left ventrcular and right ventricular dimensions reference parameters for clinical use in athletes according to gender and type of sport. *LVEDV* left ventricular end-diastolic volume, *LVESV* left ventricular end-systolic volume, *LVEF* left ventricular ejection fraction, *LVM* left ventricular mass, *RVEDV* right ventricular end-diastolic volume, *RVESV* right ventricular end-systolic volume, *RVRF* right ventricular ejection fraction, *RVM* right ventricular mass, *RWM* relative wall mass, *PM* papillary muscle

LV dimensions and LV wall thickness and LV/RV ratios reference parameters for clinical use in athletes of medium and high intensity sports (no significant differences were found between control and low intensity sport groups) are reported in Table 4, Fig. 1 and Additional file 1: Table S3, with gender cut-offs when applicable. Furthermore, reference parameters for specific sports are also provided. Overall, LVM excluding papillary muscles was 78 ± 13 g/m^2^, LV end-diastolic volume 102 ± 16 mL/m^2^, LV end-systolic volume 38 ± 9 mL/m^2^, LVEF 64 ± 6%, and LV relative wall mass excluding papillary muscles 0.75 ± 0.16 g/mL. In the multivariate analysis, in general, LV parameters were greater in men than in women, without relevant differences according to sport or age, except for a limited number of variables (Additional file 1: Table S4).

**Table 4:** Left ventricular morphology and function, wall thickness dispersion index, relative wall mass and left ventricular/right ventricular ratios reference parameters summary data (mean, 95% confidence interval) for medium and high intensity sports, with gender cut-offs when applicable, and for specific sports

	Medium and high intensity sports			Running, soccer		Cycling, rowing, triathlon	
	All	By gender					
		Males	Females	Males	Females	Males	Females
LV EDV (mL)^†‡^	186 ± 36 (116, 256)	214 ± 38 (140, 288)	159 ± 27 (106, 212)	211 ± 41 (130, 292)	150 ± 23 (105, 197)	211 ± 38 (136, 286)	159 ± 27 (106, 212)
LV EDV/BSA (mL/m^2^)^†^	102 ± 16 (72, 134)	110 ± 17 (77, 143)	95 ± 13 (70, 120)	111 ± 18 (76, 146)	95 ± 15 (66, 124)	109 ± 17 (76, 143)	93 ± 13 (68, 128)
LV ESV (mL)^*†‡^	68 ± 19 (31, 105)	78 ± 21 (37, 119)	58 ± 15 (29, 87)	76 ± 20 (37, 116)	58 ± 13 (32, 84)	79 ± 23 (34, 124)	59 ± 14 (31, 87)
LV ESV/BSA (mL/m^2^)^*†^	38 ± 9 (21, 57)	40 ± 10 (21, 59)	36 ± 8 (20, 52)	40 ± 10 (21, 59)	35 ± 7 (21, 49)	42 ± 11 (21, 65)	37 ± 6 (25, 49)
LV EF (%)*	64 ± 6 (53, 74)	64 ± 6 (53, 74)	64 ± 6 (53, 74)	64 ± 6 (52, 75)	63 ± 7 (50, 77)	63 ± 6 (51, 75)	63 ± 7 (50, 77)
LVM excluding PM (g)^†^	142 ± 28 (89, 197)	176 ± 31 (116, 236)	110 ± 18 (75, 145)	173 ± 29 (116, 231)	108 ± 19 (71, 145)	176 ± 31 (115, 237)	116 ± 18 (80, 151)
LVM excluding PM /BSA (g/m^2^)^†^	78 ± 13 (52, 103)	90 ± 14 (63, 117)	65 ± 10 (45, 86)	90 ± 14 (63, 117)	69 ± 13 (44, 94)	91 ± 14 (63, 118)	73 ± 11 (51, 95)
LV RWM excluding PM (g/mL)^†^	0.75 ± 0.16 (0.43, 1.07)	0.82 ± 0.18 (0.47, 1.17)	0.69 ± 0.13 (0.44, 0.95)	0.80 ± 0.19 (0.43, 1.17)	0.73 ± 0.11 (0.51, 0.95)	0.84 ± 0.18 (0.48, 1.20)	0.74 ± 0.12 (0.50, 0.97)
Max WT (mm)^†^	9.0 ± 1.4 (6, 12)	10.0 ± 1.5 (7, 13)	8.0 ± 1.3 (5, 11)	10.2 ± 1.5 (7, 13)	8.0 ± 0.9 (6, 10)	10.4 ± 1.6 (7, 13)	8.5 ± 1.1 (7, 11)
WTDI (mm)^†^	1.01 ± 0.21 (0.59, 1.43)	1.17 ± 0.21 (0.75, 1.59)	.85 ± 0.20 (0.45, 1.25)	1.15 ± 0.21 (0.74, 1.57)	.86 ± 0.20 (0.46, 1.25)	1.09 ± 0.23 (0.65, 1.55)	0.90 ± 0.20 (0.50, 1.30)
WT AB ratio	0.80 ± 0.07 (0.66, 0.94)	0.79 ± 0.06 (0.67, 0.91)	0.80 ± 0.07 (0.66, 0.94)	0.78 ± 0.07 (0.64, 0.92)	0.78 ± 0.07 (0.64, 0.92)	0.81 ± 0.05 (0.71, 0.91)	0.79 ± 0.04 (0.71, 0.87)
PM mass (g)^†^	5.5 ± 1.4 (2.8, 8.3)	6.3 ± 1.5 (3.5, 9.1)	4.8 ± 1.3 (2.3, 7.4)	6.2 ± 1.5 (3.3, 9.1)	4.8 ± 1.0 (2.8, 6.8)	6.3 ± 1.5 (3.3, 9.3)	5.1 ± 1.3 (2.5, 7.6)
PM mass /BSA (g/m^2^)^†^	3.0 ± 0.8 (1.5, 4.6)	3.2 ± 0.8 (1.7, 4.8)	2.8 ± 0.7 (1.5, 4.1)	3.3 ± 0.8 (1.7, 4.8)	3.0 ± 0.5 (2.0, 4.0)	3.3 ± 0.8 (1.7, 4.8)	3.3 ± 0.6 (2.1, 4.5)
LV AVPD, septal (%)	20 ± 3 (14, 26)	19 ± 3 (13, 25)	20 ± 3 (14, 26)	19 ± 3 (13, 25)	20 ± 3 (14, 26)	18 ± 4 (10, 26)	19 ± 4 (11, 27)
LV AVPD, lateral (%)^*^	19 ± 3 (13, 25)	18 ± 3 (12, 24)	19 ± 3 (13, 25)	18 ± 3 (12, 24)	19 ± 3 (13, 25)	18 ± 4 (10, 25)	19 ± 3 (13, 25)
Sphericity index, ES	2.32 ± 0.33 (1.67, 2.97)	2.34 ± 0.33 (1.68, 2.00)	2.30 ± 0.32 (1.66, 2.94)	2.30 ± 0.30 (1.70, 2.90)	2.29 ± 0.30 (1.69, 2.89)	2.40 ± 0.32 (1.67, 3.03)	2.35 ± 0.31 (1.63, 2.97)
Sphericity index, ED^*^	1.97 ± 0.20 (1.58, 2.36)	1.98 ± 0.20 (1.57, 2.36)	2.00 ± 0.20 (1.60, 2.40)	1.93 ± 0.20 (1.54, 2.33)	2.00 ± 0.17 (1.66, 2.34)	1.99 ± 0.21 (1.57, 2.40)	1.98 ± 0.20 (1.59, 2.38)
LV / RV EDV ratio	1.01 ± 0.10 (0.80, 1.20)	1.02 ± 0.10 (0.82, 1.22)	1.01 ± 0.10 (0.81, 1.21)	1.03 ± 0.11 (0.91, 1.24)	0.99 ± 0.09 (0.81, 1.17)	1.02 ± 0.10 (0.82, 1.22)	1.03 ± 0.11 (0.81, 1.24)
LV / RV ESV ratio	0.95 ± 0.17 (0.61, 1.28)	0.96 ± 0.17 (0.62, 1.30)	0.94 ± 0.17 (0.60, 1.28)	1.00 ± 0.17 (0.66, 1.33)	0.97 ± 0.17 (0.63, 1.30)	0.97 ± 0.17 (0.63, 1.30)	0.98 ± 0.17 (0.64, 1.31)
LV / RV EF ratio	1.05 ± 0.08 (0.89, 1.21)	1.03 ± 0.07 (0.89, 1.17)	1.06 ± 0.08 (0.90, 1.22)	1.03 ± 0.07 (0.89, 1.17)	1.03 ± 0.08 (0.88, 1.19)	1.04 ± 0.07 (0.90, 1.17)	1.05 ± 0.08 (0.90, 1.21)

*LV* left ventricle, *EDV* end-diastolic volume, *BSA* body surface area, *ESV* end-systolic volume, *EF* ejection fraction, *LVM* left ventricular mass, *PM* papillary muscles, *RWM* relative wall mass, *Max WT* maximum wall thickness, *WTDI* wall thickness dispersion index, *WT AB ratio* global apical to basal wall thickness ratio, *PM mass* total papillary muscle mass, *AVPD* atrioventricular plane displacement, *ES* end-systole, *ED* end-diastole, *RV* right ventricle

^*^Significant differences (p < 0.05) among age groups on multivariable analysis

^†^Significant differences (p < 0.05) between genders on multivariable analysis

^‡^Significant differences (p < 0.05) between medium and high intensity sport categories on multivariable analysis

Equally, RV dimensions reference parameters for clinical use in athletes of medium and high intensity sports are reported in Table 5, Fig. 1 and Additional file 1: Table S5, with gender and age cut-offs when applicable. Overall, RV mass was 35 ± 7 g/m^2^, RV end-diastolic volume 102 ± 17 mL/m^2^, RV end-systolic volume 40 ± 10 mL/m^2^, and RVEF 61 ± 6%. In the multivariate analysis, in general, RV parameters were greater in men than in women, without relevant differences according to sport or age, except for a limited number of variables (Additional file 1: Table S6).

**Table 5:** Right ventricular morphology and function, reference parameters summary data (mean, 95% confidence interval) for medium and high intensity sports, with gender cut-offs when applicable, and for specific sports

	Medium and high intensity sports			Running, soccer		Cycling, rowing, triathlon	
	All	By gender					
		Males	Females	Males	Females	Males	Females
RV EDV (mL)^†^	182 ± 37 (108, 255)	212 ± 38 (136, 288)	150 ± 28 (94, 206)	208 ± 38 (133, 284)	152 ± 25 (103, 202)	207 ± 35 (138, 277)	155 ± 24 (107, 202)
RV EDV/BSA (mL/m^2^)^†^	102 ± 17 (68, 136)	109 ± 18 (74, 144)	96 ± 15 (66, 125)	109 ± 18 (74, 144)	97 ± 16 (65, 128)	107 ± 17 (75, 141)	95 ± 15 (66, 125)
RV ESV (mL)^*†^	73 ± 20 (34, 112)	81 ± 21 (40, 121)	62 ± 18 (26, 98)	77 ± 18 (41, 113)	58 ± 12 (34, 82)	80 ± 19 (42, 118)	62 ± 11 (40, 84)
RV ESV/BSA (mL/m^2^)^*†^	40 ± 10 (20, 59)	42 ± 11 (20, 63)	38 ± 8 (23, 55)	41 ± 10 (21, 60)	37 ± 8 (21, 53)	43 ± 11 (21, 64)	38 ± 7 (24, 52)
RV EF (%)^*^	61 ± 6 (49, 73)	62 ± 6 (50, 73)	60 ± 5 (49, 71)	63 ± 6 (51, 75)	62 ± 5 (52, 72)	61 ± 6 (49, 73)	60 ± 5 (50, 70)
RVM (g)^†‡^	60 ± 14 (33, 89)	76 ± 15 (46, 106)	48 ± 10 (28, 68)	74 ± 14 (47, 102)	47 ± 8 (31, 62)	76 ± 15 (46, 105)	49 ± 6 (37, 61)
RVM/BSA (g/m^2^)^†^	35 ± 7 (21, 48)	40 ± 8 (24, 55)	30 ± 5 (21, 40)	39 ± 7 (25, 53)	30 ± 6 (18, 42)	40 ± 8 (24, 55)	30 ± 5 (20, 40)
RV RWM (g/mL)^†^	0.35 ± 0.07 (0.21, 0.48)	0.37 ± 0.07 (0.23, 0.51)	0.33 ± 0.05 (0.23, 0.42)	0.35 ± 0.07 (0.21, 0.49)	0.31 ± 0.07 (0.27, 0.45)	0.37 ± 0.09 (0.20, 0.54)	0.31 ± 0.05 (0.21, 0.41)
RV WT (mm)^†^	3.8 ± 0.7 (2.5, 5.1)	4.0 ± 0.7 (2.6, 5.3)	3.5 ± 0.5 (2.6, 4.5)	0.40 ± 0.07 (0.26, 54)	0.35 ± 0.05 (0.25, 0.45)	0.41 ± 0.08 (0.25, 0.56)	0.34 ± 0.05 (0.25, 0.45)
RV AVPD, lateral (%)	25 ± 5 (16, 36)	26 ± 5 (16, 36)	25 ± 5 (15, 35)	26 ± 5 (16, 36)	23 ± 4 (15, 31)	24 ± 5 (14, 34)	22 ± 5 (13, 33)
RV sphericity index, ES	2.38 ± 0.49 (1.40, 3.36)	2.35 ± 0.49 (1.37, 3.33)	2.39 ± 0.50 (1.39, 3.39)	2.35 ± 0.47 (1.41, 3.29)	2.39 ± 0.36 (1.67, 3.41)	2.20 ± 0.41 (1.39, 3.01)	2.25 ± 0.31 (1.63, 2.88)
RV sphericity index, ED	2.45 ± 0.44 (1.67, 3.33)	2.44 ± 0.44 (1.66, 3.32)	2.46 ± 0.45 (1.66, 3.35)	2.46 ± 0.39 (1.68, 3.24)	2.48 ± 0.41 (1.66, 3.30)	2.31 ± 0.38 (1.57, 3.07)	2.41 ± 0.40 (1.61, 3.21)

*RV* right ventricle, *EDV* end-diastolic volume, *BSA* body surface area, *ESV* end-systolic volume, *EF* ejection fraction, *RVM* right ventricular mass, *RWM* relative wall mass, *WT* free wall thickness (measured in segment 7), *AVPD* atrioventricular plane displacement, *ES* end-systole, *ED* end-diastole

^*^Significant differences (p < 0.05) among age groups on multivariable analysis

^†^Significant differences (p < 0.05) between genders on multivariable analysis

^‡^Significant differences (p < 0.05) among sport categories (medium and high intensity) on multivariable analysis

## Discussion

In our study, extensive reference CMR values of Caucasian athletes compared with a control group are provided. In addition, data were analyzed according to sport category with respect to type of sport, age and gender.

Although sports practice promotes metabolic, functional and physical benefits, some athletes may develop adverse outcomes, including sudden cardiac death [20, 21]. On the other hand, high intensity exercise stimulates changes in cardiac structure and function that translates into a physiological adaptation to physical activity, the “Athlete's Heart” [22]. As a result, it is mandatory the use of diagnostic tools that may be of help to distinguish between physiological and pathological changes on heart as this may have relevant prognostic consequences [23–25]. In this context, although the ECG and echocardiogram are considered the first step in the diagnostic approach to identify the structural adaptative changes observed in the hearts of highly trained athletes, CMR provides a higher volumetric accuracy to differentiate normal adaptive athlete's heart from mild or initial expression forms of left- and right-heart side cardiomyopathies [26–28]. In fact, the Society for Cardiovascular Magnetic Resonance supports a class II recommendation for the use of CMR in the diagnosis of athlete’s heart [29]. Unfortunately, CMR reference values from athletes are scarce [30–32, 35]. Although the number of patients included in our study was inferior to those reported with echocardiography, it should be considered that the reproducibility of CMR is higher and, consequently, the number of subjects required is significantly lower [33]. Therefore, our data provided relevant information that may be very valuable for their use clinical practice.

Different studies have reported that cardiac changes induced by exercise depend on the type of training. Thus, although normal systolic and diastolic cardiac functions remain stable in the athlete's heart, pure endurance and strength training would promote different effects on cardiac remodeling [1, 34]. Still, training programs usually include both types of exercise, in a variable proportion, so in real life cardiovascular changes in response to exercise are not dichotomous. In our study, athletes with both type of training were included. In addition, control and athlete groups were well balanced regarding age, sex, anthropometric characteristics and medical history. All of this suggests that our data could be applied to the whole athlete population.

In our study, LVM and RV mass, end-diastolic and end-systolic volumes, as well as wall thickness were not only higher in athletes than in the control group, but also these numbers gradually increased with the intensity of sport. Of note, LVEF and RVEF were similar in both groups, without a significant impact according to sport category or type of sport, supporting the generalizability of the results. These data are in line with previous studies that have observed that increases in LV and RV volumes and mass are related to the amount of training rather than the sporting discipline [31, 35–37]. However, data from low intensity sports and control group were similar, what is important in clinical practice in order to define normal CMR values. On the other hand, it should be emphasized that we analyzed many dimensions and function parameters and ratios, providing a more complete CMR analysis than previously reported in highly trained athletes [31]. In fact, we provided many important parameters that have not been previously published (i.e. segmental parietal thicknesses, some ratios, desynchrony index, etc.) and that may have an important impact in the management of patients in clinical practice. For example, in contrast to previous studies with athletes, we measured the size of papillary muscles that are not well defined with echocardiography. Therefore, our data are important to differentiate normal values in athletes from those of patients with Fabry´s disease or hypertrophic cardiomyopathy [38, 39]. Additionally, we also measured the maximum wall thickness, the wall thickness dispersion index, and apical/basal wall thickness ratios, that can be helpful parameters for the early diagnosis of mild apical hypertrophic cardiomyopathy [40].

Morphological, functional, and electrical changes of the cardiac chambers induced by exercise do not only depend on type or intensity of training, but also on age and sex [34]. Thus, a recent study that investigated the impact of sex, age, body size, sports type and training volume on cardiac adaptation in healthy athletes with CMR showed that male athletes had higher LV and RV volumes and masses in both adult and adolescent groups compared with women [41]. In our study, the multivariate analysis showed that in general, LV and RV parameters were greater in men than in women, without relevant differences according to sport or age in the majority of variables. In fact, it has been reported that training intensity, rather than age has a relevant impact on changes in cardiac parameters [36]. As a result, LV and RV dimensions reference parameters were provided not only in the general athlete population, but also according to gender and age when applicable.

Providing a quantitative evaluation of ventricular chambers is important and this may allow differentiating between pathology and normal conditions, establishing the severity of pathologies and monitoring changes during the follow-up under therapy [30]. However, to date, only some reference ranges for CMR have been provided for athletes, but limited to some particular conditions (i.e. males, right heart, etc.) [31, 42]. Thus, current information about CMR values in athletes come from relatively small studies with homogeneous sporting disciplines or with suspected cardiomyopathy [32]. A recent meta-analysis of studies involving CMR in apparently adult healthy competitive athletes have proposed normal values for biventricular size and function [31]. As in our case, volumes and mass were greater in athletes than in the general population, and biventricular function was not significantly affected by training volume [31]. However, this meta-analysis was limited to male athletes and substantial heterogeneity were found between studies [31]. As a result, new data that may provide normal values for cardiac dimensions in athletes engaging in a variety of sports rather than using standard upper limits derived from the general population are warranted [32]. In this context, our study provided LV and RV dimensions and functional parameters as well as LV/RV ratios reference parameters for athletes of moderate and high cardiovascular demand sports that could be very helpful in the management of this population. Furthermore, data were provided including/excluding papillary muscles, and reference numbers about wall thickness were also given. In addition, numbers were provided according to gender and age when applicable.

### Limitations

Our study has several limitations. First, although the overall sample size was enough to respond the main objective of the study, when analyzing some subgroups of patients (i.e. female athletes), large error in the estimates could occur, causing wider confidence intervals. In addition, not all types of training were equally represented, and should be specifically analyzed in further researches. As previously commented, cardiac remodeling is influenced by age. Unfortunately, only in some specific variables reference values could be provided according to age. Therefore, when considering reference values, training regimen, intensity, years of training, gender and age should be considered. On the other hand, late gadolinium enhancement was not performed, but this was not the scope of the study. Finally, as this study was performed in European Caucasian subjects, the current results cannot be necessarily applied to athletes of other races or origin and further CMR studies are needed in these populations.

## Conclusions

CMR is a relevant tool in the evaluation of athlete’s heart and may be considered for a most comprehensive approach in the management of athletes to assess for pathology. LV and RV masses, volumes, and wall thickness were higher in athletes than in the control group, but also these numbers gradually increased with the intensity of sport. LVEF and RVEF were independent of the intensity of activity. Specific CMR reference ranges for athletes are provided and can be used as reference levels in this population, rather than the standard upper limits used for the general population to establish normality and exclude cardiomyopathy.

## Supplementary Information

**Additional file 1: Table S1.** Additional LV morphology parameters, LV segmental wall thickness and LV ratios for ventricular parameters in controls and athletes classified with respect to sport category. **Table S2.** Additional RV morphology parameters in controls and athletes classified with respect to sport category. **Table S3.** Additional LV morphology parameters, LV segmental wall thickness and LV ratios summary data (mean, 95% confidence interval) for athletes of medium and high intensity sports, with gender cut-offs when applicable. **Table S4.** Effect size of sport type (medium and high intensity), gender and age on LV parameters, LV wall thickness, LV and LV/RV ratios on multivariate analysis. **Table S5.** Additional RV dimensions reference parameters summary data (mean, 95% confidence interval) for athletes of medium and high intensity sports, with gender cut-offs when applicable. **Table S6.** Effect size of sport type (medium and high intensity), gender and age on RV parameters on multivariate analysis.

## Data Availability

Original are available under request.

## Abbreviations

AV: Atrioventricular
AVPD: Atrioventricular plane descent
CMR: Cardiovascular magnetic resonance
ECG: Electrocardiogram
EDV: End-diastolic volume
EF: Ejection fraction
ESV: End-systolic volume
LV: Left ventricle/left ventricular
LVEF: Left ventricular ejection fraction
LVM: Left ventricular mass
RV: Right ventricle/right ventricular
RVEF: Right ventricular ejection fraction
RVM: Relative ventricular mass
RWM: Relative wall mass

## References

[1] Pluim B, Zwinderman A, van der Laarse A, van der Wall E, Pluim BM, Zwinderman AH (2000). The athlete’s heart: a meta-analysis of cardiac structure and function. Circulation.

[2] Maron BJPA (2006). The heart of trained athletes. Circulation.

[3] Maron B (2005). Distinguishing hypertrophic cardiomyopathy from athlete’s heart: a clinical problem of increasing magnitude and significance. Heart.

[4] Millar LM, Fanton Z, Finocchiaro G, Sanchez-Fernandez G, Dhutia H, Malhotra A (2020). Differentiation between athlete’s heart and dilated cardiomyopathy in athletic individuals. Heart.

[5] Bauce B, Frigo G, Michieli P, Basso C, Folino A, Rigato I (2010). Differences and similarities between arrhythmogenic right ventricular cardiomyopathy and athlete’s heart adaptations. Br J Sports Med.

[6] Harmon KG, Asif IM, Maleszewski JJ, Owens DS, Prutkin JM, Salerno JC (2015). Incidence, cause, and comparative frequency of sudden cardiac death in national collegiate athletic association athletes: a decade in review. Circulation.

[7] Schnell F, Riding N, O'Hanlon R, Axel Lentz P, Donal E, Kervio G (2015). Recognition and significance of pathological T-wave inversions in athletes. Circulation.

[8] Doherty JU, Kort S, Mehran R, Schoenhagen P, Soman P, Dehmer GJ (2019). ACC/AATS/AHA/ASE/ASNC/HRS/SCAI/SCCT/SCMR/STS 2019 appropriate use criteria for multimodality imaging in the assessment of cardiac structure and function in nonvalvular heart disease: a report of the American College of Cardiology Appropriate Use Criteria. J Am Coll Cardiol.

[9] Pelliccia A, Sharma S, Gati S (2021). 2020 ESC Guidelines on sports cardiology and exercise in patients with cardiovascular disease [published correction appears in Eur Heart J. 2021 Feb 1;42(5):548-549]. Eur Heart J..

[10] Philipp B, Günther S, Lutz L, Axel R, Nadine K, Hashim A-K (2016). Right and left ventricular function and mass in male elite master athletes. Circulation.

[11] Williams B, Mancia G, Spiering W, Agabiti Rosei E, Azizi M, Burnier M (2018). 2018 ESC/ESH Guidelines for the management of arterial hypertension: the Task Force for the management of arterial hypertension of the European Society of Cardiology (ESC) and the European Society of Hypertension (ESH). Eur Heart J.

[12] Cosentino F, Grant PJ, Aboyans V, Bailey CJ, Ceriello A, Delgado V (2019). 2019 ESC Guidelines on diabetes, pre-diabetes, and cardiovascular diseases developed in collaboration with the EASD: the Task Force for diabetes, pre-diabetes, and cardiovascular diseases of the European Society of Cardiology (ESC) and the European Associ. Eur Heart J.

[13] Mach F, Baigent C, Catapano AL, Koskinas KC, Casula M, Badimon L (2019). 2019 ESC/EAS Guidelines for the management of dyslipidaemias: lipid modification to reduce cardiovascular risk: The Task Force for the management of dyslipidaemias of the European Society of Cardiology (ESC) and European Atherosclerosis Society (EAS). Eur Heart J.

[14] Sharma S, Drezner JA, Baggish A, Papadakis M, Wilson MG, Prutkin JM (2018). International recommendations for electrocardiographic interpretation in athletes. Eur Heart J.

[15] Mosteller R (1987). Simplified calculation of body-surface area. N Engl J Med.

[16] Kramer CM, Barkhausen J, Bucciarelli-Ducci C, Flamm SD, Kim RJ, Nagel E (2020). Standardized cardiovascular magnetic resonance imaging (CMR) protocols: 2020 update. J Cardiovasc Magn Reson.

[17] Cerqueira MD, Weissman NJ, Dilsizian V, Jacobs AK, Kaul S, Laskey WK (2002). Standardized myocardial segmentation and nomenclature for tomographic imaging of the heart. Circulation.

[18] Prati G, Vitrella G, Allocca G, Muser D, Buttignoni SC, Piccoli G (2015). Right ventricular strain and dyssynchrony assessment in arrhythmogenic right ventricular cardiomyopathy: cardiac magnetic resonance feature-tracking study. Circ Cardiovasc Imaging.

[19] Kono T, Sabbah H, Stein P, Brymer J, Khaja F (1991). Left ventricular shape as a determinant of functional mitral regurgitation in patients with severe heart failure secondary to either coronary artery disease or idiopathic dilated cardiomyopathy. Am J Cardiol.

[20] Pelliccia A, Sharma S, Gati S, Bäck M, Börjesson M, Caselli S (2021). 2020 ESC Guidelines on sports cardiology and exercise in patients with cardiovascular disease. Eur Heart J.

[21] de Gregorio C, Di Nunzio D, Di Bella G (2018). Athlete's heart and left heart disease. Adv Exp Med Biol.

[22] Brosnan MJ, Rakhit D (2018). Differentiating athlete's heart from cardiomyopathies—the left side. Heart Lung Circ.

[23] Gastl M, Lachmann V, Christidi A, Janzarik N, Veulemans V, Haberkorn S (2021). Cardiac magnetic resonance T2 mapping and feature tracking in athlete's heart and HCM. Eur Radiol.

[24] Vilades D, Garcia-Moll X, Gomez-Llorente M, Pujadas S, Ferrero-Gregori A, Doñate T (2021). Differentiation of athlete's heart and hypertrophic cardiomyopathy by the fractal dimension of left ventricular trabeculae. Int J Cardiol.

[25] Caruso MR, Garg L, Martinez MW (2020). Cardiac imaging in the athlete: shrinking the "Gray Zone". Curr Treat Options Cardiovasc Med.

[26] Abulí M, de la Garza MS, Sitges M (2020). Differentiating athlete's heart from left ventricle cardiomyopathies. J Cardiovasc Transl Res.

[27] D'Ascenzi F, Anselmi F, Mondillo S, Finocchiaro G, Caselli S, Garza MS (2021). The use of cardiac imaging in the evaluation of athletes in the clinical practice: a survey by the Sports Cardiology and Exercise Section of the European Association of Preventive Cardiology and University of Siena, in collaboration with the European Association of Cardiovascular Imaging, the European Heart Rhythm Association and the ESC Working Group on Myocardial and Pericardial Diseases. Eur J Prev Cardiol.

[28] Maestrini V, Torlasco C, Hughes R, Moon JC (2020). Cardiovascular magnetic resonance and sport cardiology: a growing role in clinical dilemmas. J Cardiovasc Transl Res.

[29] Leiner T, Bogaert J, Friedrich MG, Mohiaddin R, Muthurangu V, Myerson S (2020). SCMR Position Paper (2020) on clinical indications for cardiovascular magnetic resonance. J Cardiovasc Magn Reson.

[30] Kawel-Boehm N, Hetzel SJ, Ambale-Venkatesh B, Captur G, Francois CJ, Jerosch-Herold M (2020). Reference ranges ("normal values") for cardiovascular magnetic resonance (CMR) in adults and children: 2020 update. J Cardiovasc Magn Reson.

[31] D'Ascenzi F, Anselmi F, Piu P, Fiorentini C, Carbone SF, Volterrani L (2019). Cardiac magnetic resonance normal reference values of biventricular size and function in male athlete's heart. JACC Cardiovasc Imaging.

[32] Sharma S, Malhotra A (2019). Cardiac magnetic resonance imaging in athletes: acquiring the bigger picture. JACC Cardiovasc Imaging.

[33] Fábián A, Ujvári A, Tokodi M, Lakatos BK, Kiss O, Babity M (2022). Biventricular mechanical pattern of the athlete's heart: comprehensive characterization using 3D echocardiography. Eur J Prev Cardiol..

[34] D'Andrea A, Formisano T, Riegler L, Scarafile R, America R, Martone F (2017). Acute and chronic response to exercise in athletes: the "supernormal heart". Adv Exp Med Biol.

[35] Prakken NH, Velthuis BK, Teske AJ, Mosterd A, Mali WP, Cramer MJ (2010). Cardiac MRI reference values for athletes and nonathletes corrected for body surface area, training hours/week and sex. Eur J Cardiovasc Prev Rehabil.

[36] Prakken NH, Cramer MJ, Teske AJ, Arend M, Mali WP, Velthuis BK (2011). The effect of age in the cardiac MRI evaluation of the athlete's heart. Int J Cardiol.

[37] Luijkx T, Cramer MJ, Prakken NH, Buckens CF, Mosterd A, Rienks R (2012). Sport category is an important determinant of cardiac adaptation: an MRI study. Br J Sports Med.

[38] Al-Arnawoot A, O'Brien C, Karur GR, Nguyen ET, Wasim S, Iwanochko RM (2021). Clinical significance of papillary muscles on left ventricular mass quantification using cardiac magnetic resonance imaging: reproducibility and prognostic value in Fabry disease. J Thorac Imaging.

[39] Dohy Z, Szabo L, Toth A, Czimbalmos C, Horvath R, Horvath V (2021). Prognostic significance of cardiac magnetic resonance-based markers in patients with hypertrophic cardiomyopathy. Int J Cardiovasc Imaging.

[40] Yin Y, Hu W, Zhang L, Wu D, Yang C, Ye X (2022). Clinical, echocardiographic and cardiac MRI predictors of outcomes in patients with apical hypertrophic cardiomyopathy. Int J Cardiovasc Imaging.

[41] Csecs I, Czimbalmos C, Toth A, Dohy Z, Suhai IF, Szabo L (2020). The impact of sex, age and training on biventricular cardiac adaptation in healthy adult and adolescent athletes: cardiac magnetic resonance imaging study. Eur J Prev Cardiol.

[42] D'Ascenzi F, Pelliccia A, Solari M (2017). Normative reference values of right heart in competitive athletes: a systematic review and meta-analysis. J Am Soc Echocardiogr.

