# Supplementary material for: Reference ventricular dimensions and function parameters by cardiovascular magnetic resonance in highly trained Caucasian athletes
Source: J Cardiovasc Magn Reson. 2023 Feb 9;25:12. doi: 10.1186/s12968-023-00910-7 (PMC9909951; doi:10.1186/s12968-023-00910-7)
Supplement: Supplementary file 1 — Additional file 1: Table S1. Additional LV morphology parameters, LV segmental wall thickness and LV ratios for ventricular parameters in controls and athletes classified with respect to sport category. Table S2. Additional RV morphology parameters in controls and athletes classified with respect to sport category. Table S3. Additional LV morphology parameters, LV segmental wall thickness and LV ratios summary data (mean, 95% confidence interval) for athletes of medium and high intensity sports, with gender cut-offs when applicable. Table S4. Effect size of sport type (medium and high intensity), gender and age on LV parameters, LV wall thickness, LV and LV/RV ratios on multivariate analysis. Table S5. Additional RV dimensions reference parameters summary data (mean, 95% confidence interval) for athletes of medium and high intensity sports, with gender cut-offs when applicable. Table S6. Effect size of sport type (medium and high intensity), gender and age on RV parameters on multivariate analysis. [file 12968_2023_910_MOESM1_ESM.docx]

**Additional file 1**

**Table S1. Additional LV morphology parameters, LV segmental wall thickness and LV ratios for ventricular parameters in controls and athletes classified with respect to sport category**

|  | **Control** | **Low intensity** | **Medium intensity** | **High intensity** | **P** |  | **Control** | **Low intensity** | **Medium intensity** | **High intensity** | **P** |
| --- | --- | --- | --- | --- | --- | --- | --- | --- | --- | --- | --- |
| **LV EDV/h (mL/m)** | 83 ± 16 ^‡§^ | 82 ± 11 ^‡§^ | 107 ± 18 ^*†§^ | 116 ± 22 ^*†‡^ | <.001 | **WT, basal anterior segment (mm)** | 7.4 ± 1.7 ^§^ | 7.6 ± 1.8 | 7.7 ± 1.6 ^*^ | 8.3 ± 1.7 ^*^ | <.05 |
| **LV EDV/ h^2.7^ (g/m^2.7^)** | 25 ± 5 ^‡§^ | 24 ± 3 ^‡§^ | 30 ± 4 ^*†^ | 31 ± 5^*†^ | <.001 | **WT, basal anteroseptal segment (mm)** | 7.8 ± 1.9 ^‡§^ | 8.4 ± 1.5 | 8.8 ± 1.9 ^*^ | 9.3 ± 1.9 ^*^ | <.001 |
| **LV ESV/h (mL/m)** | 28 ± 10 ^‡§^ | 28 ± 6 ^‡§^ | 38 ± 10 ^*†§^ | 43 ± 12 ^*†§^ | <.001 | **WT, basal inferoseptal segment (mm)** | 7.2 ± 1.2 ^‡§^ | 7.8 ± 1.6 | 8.0 ± 1.4 ^*^ | 8.5 ± 1.6 ^*^ | <.001 |
| **LV ESV/ h^2.7^ (g/m^2.7^)** | 8.4 ± 2.8 ^‡§^ | 8.2 ± 2 ^‡§^ | 10.6 ± 2.5 ^*†^ | 11.5 ± 2.8 ^*†^ | <.001 | **WT, basal inferior segment (mm)** | 7.0 ± 1.2 ^‡§^ | 7.8 ± 1.8 | 7.9 ± 1.6 ^*^ | 8.2 ± 1.6 ^*^ | <.05 |
| **LVM excluding PM /h (g/m)** | 71 ± 17 ^‡§^ | 69 ± 23 ^‡§^ | 84 ± 20 ^*†§^ | 92 ± 19 ^*†‡^ | <.001 | **WT, basal inferolateral segment (mm)** | 7.2 ± 1.2 ^‡§^ | 8.0 ± 1.9 | 8.4 ± 1.8 ^*^ | 8.6 ± 1.9 ^*^ | <.001 |
| **LVM excluding PM/h^2.7^ (g/m^2.7^)** | 21 ± 4 ^‡§^ | 20 ± 6 ^‡§^ | 24 ± 5 ^*†^ | 24 ± 5 ^*†^ | <.001 | **WT, basal anterolateral segment (mm)** | 7.1 ± 1.1 ^‡§^ | 7.4 ± 1.9 | 7.8 ± 1.6 ^*^ | 8.1 ± 1.6 ^*^ | <.05 |
| **PM, anterosuperior (g)** | 2.6 ± .8 ^§^ | 2.6 ± .8 | 3.0 ± 1.0 | 3.4 ± 1 ^*^ | <.05 | **WT, mid anterior segment (mm)** | 6.4 ± 1.1 ^‡§^ | 6.4 ± 1.4 | 7.1 ± 1.4 ^*^ | 7.1 ± 1.5 ^*^ | <.05 |
| **PM, anterosuperior/BSA (g/m^2^)** | 1.4 ± .4 ^‡§^ | 1.5 ± .5 | 1.7 ± .6^*^ | 1.8 ± .5^*^ | <.05 | **WT, mid anteroseptal segment (mm)** | 6.9 ± 1.3 ^‡§^ | 7.8 ± 2.1 | 8.0 ± 1.6 ^*^ | 8.3 ± 1.8 ^*^ | <.001 |
| **PM, posteroinferior (g)** | 2.0 ± .6 ^‡§^ | 2.1 ± .4 | 2.4 ± .8 ^*§^ | 2.8 ± 1 ^*‡^ | <.001 | **WT, mid inferoseptal segment (mm)** | 7.3 ± 1.3 ^‡§^ | 7.9 ± 1.7 | 8.3 ± 1.8 ^*^ | 8.8 ± 1.6 ^*^ | <.001 |
| **PM, posteroinferior/BSA (g/m^2^)** | 1.1 ± .3 ^‡§^ | 1.3 ± .2 | 1.3 ± .4 ^*^ | 1.4 ± .5 ^*^ | <.001 | **WT, mid inferior segment (mm)** | 7.0 ± 1.3 ^‡§^ | 7.0 ± 1.8 | 7.5 ± 1.4 ^*^ | 7.8 ± 1.6 ^*^ | <.001 |
| **PM mass/LVM excluding PM (%)** | 3.7 ± .98 | 4.0 ± 1.0 | 3.8 ± 1.1 | 3.8 ± .8 | NS | **WT, mid inferolateral segment (mm)** | 6.7 ± 1.1 ^‡§^ | 7.0 ± 1.9 | 7.5 ± 1.6 ^*^ | 7.9 ± 1.6 ^*^ | <.001 |
| **LVM including PM (g)** | 123 ± 31^‡§^ | 118 ± 30^‡§^ | 153 ± 41 ^*‡§^ | 173 ± 39^*†‡^ | <.001 | **WT, mid anterolateral segment (mm)** | 6.6 ± 1.1 ^‡§^ | 6.8 ± 1.6 | 7.1 ± 1.6 ^*^ | 7.5 ± 1.6 ^*^ | < .05 |
| **LVM/BSA including PM (g/m^2^)** | 68 ± 14 ^‡§^ | 68 ± 18 ^‡§^ | 85 ± 18 ^*†^ | 89 ± 16^*†^ | <.001 | **WT, apical anterior segment (mm)** | 5.6 ± 1.1 ^‡§^ | 6.0 ± 1.2 | 6.6 ± 1.3 ^*^ | 6.7 ± 1.5 ^*^ | <.001 |
| **LVM/h including PM (g/m)** | 72 ± 17 ^‡§^ | 67 ± 20 ^‡§^ | 87 ± 20 ^*†§^ | 96 ± 18 ^*†‡^ | <.05 | **WT, apical septal segment (mm)** | 5.9 ± 1.1 ^‡§^ | 6.4 ± 1.7 | 6.9 ± 1.3 ^*^ | 7.2 ± 1.5 ^*^ | <.001 |
| **LVM/h^2.7^ including PM (g/m^2.7^)** | 21 ± 5 ^‡§^ | 20 ± 6 ^‡§^ | 24 ± 5 ^*†^ | 26 ± 5 ^*†^ | <.05 | **WT, apical inferior segment (mm)** | 5.9 ± 1.2 ^‡§^ | 6.2 ± 1.4 | 6.8 ± 1.5 ^*^ | 7.0 ± 1.6 ^*^ | <.001 |
| **LV RWM including PM (g/mL)** | .87 ± .15 ^§^ | .83 ± .23 | .81 ± .17 | .82 ± .17 ^*^ | <.05 | **WT, apical lateral segment (mm)** | 5.8 ± 1.0 ^‡§^ | 6.0 ± 1.7 | 6.5 ± 1.3 ^*^ | 6.7 ± 1.5 ^*^ | <.001 |
| **PM mass /LVM including PM (%)** | 3.8 ± 1.1 | 3.9 ± 1.2 | 3.8 ± 1.1 | 3.8 ± 1.0 | NS | **LV anterior WT, AB ratio** | .80 ± .10 | .79 ± .09 | .81 ± .09 | .80 ± .10 | NS |
| **LV max/min WT ratio** | 1.62 ± .19 | 1.64 ± .44 | 1.64 ± .33 | 1.62 ± .33 | NS | **LV septal WT, AB ratio** | .80 ± .09 | .78 ± .09 | .78 ± .10 | .78 ± .11 | NS |
| **LV max WT/EDV (mm/mL)** | .06 ± .02 ^‡§^ | .06 ± .02 ^‡§^ | .05 ± .01 ^*†^ | .05 ± .02 ^*†^ | <.05 | **LV inferior WT, AB ratio** | .81 ± .10 | .78 ± .11 | .80 ± .11 | .81 ± .10 | NS |
|  |  |  |  |  |  | **LV lateral WT, AB ratio** | .82 ± .11 | .81 ± .10 | .80 ± .11 | .80 ± .11 | NS |

LV, left ventricle; LVM: left ventricular mass; BSA, body surface area; h, height; PM, papillary muscle; WT, wall thickness; max, maximum; EDV, end-diastolic volume; ESV, end-systolic volume; RWM, relative wall mass; AB, apical/basal.

Post-Hoc analysis: *, significant differences with “Control” group; †, significant differences with “Low intensity” sport group; ‡, significant differences with “Medium intensity” sport group; §, significant differences with “High intensity” sport group.

**Table S2. Additional RV morphology parameters in controls and athletes classified with respect to sport category**

|  | **Control** | **Low intensity** | **Medium intensity** | **High intensity** | **P** |
| --- | --- | --- | --- | --- | --- |
| RVM/h (g/m) | 22 ± 5 ^†‡§^ | 32 ± 6 ^*§^ | 37 ± 9 ^*§^ | 41 ± 9 ^*†‡^ | <.001 |
| RVM/h^2.7^ (g/m^2.7^) | 6.3 ± 1.4 ^†‡§^ | 9.6 ± 1.7 ^*^ | 10.4 ± 2.2 ^*^ | 10.8 ± 2.2 ^*^ | <.001 |
| RV EDV/h (mL/m) | 85 ± 17 ^‡§^ | 85 ± 15 ^‡§^ | 106 ± 19 ^*†^ | 114 ± 21 ^*†^ | <.001 |
| RV EDV /h^2.7^ (mL/m^2.7^) | 25 ± 6 ^‡§^ | 25 ± 4 ^‡§^ | 30 ± 5 ^*†^ | 31 ± 5 ^*†^ | <.001 |
| RV ESV/h (mL/m) | 32 ± 10 ^‡§^ | 33 ± 8 ^‡§^ | 42 ± 10 ^*†^ | 45 ± 12 ^*†^ | <.001 |
| RV ESV / h^2.7^ (mL/m^2.7^) | 9.4 ± 2.9 ^‡§^ | 9.5 ± 2.3 ^§^ | 11.7 ± 2.7 ^*^ | 12.0 ± 3.0 ^*†^ | <.001 |

RV, right ventricle; RVM, right ventricular mass; EDV, end-diastolic volume; BSA, body surface area; h, height; ESV, end-systolic volume.

Post-Hoc analysis: *, significant differences with “Control” group; †, significant differences with “Low intensity” sport group; ‡, significant differences with “Medium intensity” sport group; §, significant differences with “High intensity” sport group.

**Table S3. Additional LV morphology parameters, LV segmental wall thickness and LV ratios summary data (mean, 95% confidence interval) for athletes of medium and high intensity sports, with gender cut-offs when applicable**

|  | **All** | **By gender** | |  | **All** | **By gender** | |
| --- | --- | --- | --- | --- | --- | --- | --- |
|  |  | **Males** | **Females** |  |  | **Males** | **Females** |
| LV EDV/h (mL/m) † | 105 ± 17  (71, 138) | 118 ± 19  (80 , 155) | 94 ± 13  (68 , 120) | LV WT, basal anterior segment (mm) † | 7.8 ± 1.4  (5.0 , 10.6) | 8.7 ± 1.4  (5.9 , 11.5) | 6.7 ± 1.1  (4.5 , 8.9) |
| LV EDV/ h^2.7^ (g/m^2.7^) † | 30 ± 5  (21 , 39) | 32 ± 5  (22 , 41) | 28 ± 4  (20 , 36) | LV WT, basal anteroseptal segment (mm) * † | 9.0 ± 1.5  (6.0 , 12.0) | 9.7 ± 1.6  (6.5, 12.9) | 7.3 ± 1.1  (5.2, 9.5) |
| LV ESV/h (mL/m) †‡ | 40 ± 10  (20 , 59) | 43 ± 11  (21 , 64) | 35 ± 8  (19, 51) | LV WT, basal inferoseptal segment (mm) † | 8.0 ± 1.2  (6.6 , 10.5) | 8.8 ± 1.2  (6.4 , 11.2) | 7.0 ± .9  (5.2 , 8.8) |
| LV ESV/ h^2.7^ (g/m^2.7^) † | 11 ± 3  (6 , 16) | 12 ± 3  (6 , 17) | 10 ± 2  (6 , 14) | LV WT, basal inferior segment (mm) † | 7.7 ± 1.2  (5.3 , 10.1) | 8.6 ± 1.2  (6.2 , 11.0) | 6.7 ± 1.0  (4.7 , 8.7) |
| LVM excluding PM /h (g/m) † | 82 ± 14  (55 , 109) | 97 ± 15  (67 , 126) | 65 ± 10  (45 , 84) | LV WT, basal inferolateral segment (mm) † | 8.1 ± 1.4  (5.3 , 10.9) | 9.1 ± 1.5  (6.1 , 12.1) | 6.9 ± 1.2  (4.5 , 9.3) |
| LVM excluding PM /h^2.7^ (g/m^2.7^) † | 23 ± 4  (15 , 30) | 26 ± 4  (18 , 34) | 20 ± 3  (14 , 26) | LV WT, basal anterolateral segment (mm) † | 7.6 ± 1.3  (5.1 , 10.3) | 8.4 ± 1.4  (5.6 , 11.2) | 6.6 ± 1.1  (4.4 , 8.8) |
| PM, anterosuperior (g) † | 3.0 ± .9  (1.2 , 4.7) | 3.5 ± 1  (1.5 , 5.4) | 2.5 ± .7  (1.2 , 3.9) | LV WT, mid anterior segment (mm) † | 7.4 ± 1.2  (5.0 , 9.7) | 7.5 ± 1.2  (5.1 , 9.9) | 6.4 ± 1.1  (4.2 , 8.6) |
| PM, anterosuperior/BSA (g/m^2^) † | 1.7 ± .5  (.7 , 2.6) | 1.8 ± .5  (.8 , 2.7) | 1.5 ± .4  (.8 , 2.4) | LV WT, mid anteroseptal segment (mm) † | 7.7 ± 1.4  (5.0 , 10.5) | 8.7 ± 1.5  (5.7 , 11.7) | 6.8 ± 1.1  (4.6 , 9.0) |
| PM, posteroinferior (g) † | 2.5 ± .9  (.8 , 4.2) | 2.8 ± .9  (1.1 , 4.5) | 2.1 ± .7  (.8 , 3.5) | LV WT, mid inferoseptal segment (mm) † | 8.1 ± 1.4  (5.4 , 10.9) | 9.2 ± 1.5  (6.3 , 12.2) | 7.0 ± 1.0  (5.0 , 9.0) |
| PM, posteroinferior/BSA (g/m^2^) † | 1.4 ± .4  (.5 , 2.2) | 1.4 ± .4  (.6 , 2.2) | 1.3 ± .4  (.6 , 2.1) | LV WT, mid inferior segment (mm) † | 7.2 ± 1.2  (4,8 , 9.6) | 8.3 ± 1.3  (5.7 , 10.9) | 6.4 ± 1.0  (4.4 , 8.4) |
| PM mass /LVM excluding PM (%) † | 3.7 ± 1.0  (1.8 , 5.7) | 3.7 ± 1.0  (1.7 , 5.6) | 4.2 ± 1.1  (2.0, 6.3) | LV WT, mid inferolateral segment (mm) † | 7.3 ± 1.4  (4.5 , 10.0) | 8.2 ± 1.4  (5.4 , 11.0) | 6.4 ± 1.2  (4.0 , 8.8) |
| LVM including PM (g) † | 149 ± 29  (91 , 206) | 182 ± 30  (122 , 242) | 119 ± 19  (81 , 157) | LV WT, mid anterolateral segment (mm) † | 7.0 ± 1.3  (4.5 , 9.6) | 7.9 ± 1.4  (5.1 , 10.7) | 6.0 ± 1.2  (4.6 , 8.4) |
| LVM /BSA including PM (g/m^2^) † | 82 ± 14  (54 , 110) | 94 ±15  (65 , 123) | 71 ± 11  (48 , 93) | LV WT, apical anterior segment (mm) † | 6.3 ± 1.1  (4.1 , 8.5) | 7.0 ± 1.2  (4.6 , 9.4) | 6.0 ± 1.0  (4.0 , 8.0) |
| LVM /h including PM (g/m) † | 85 ± 15  (55 , 115) | 100 ± 16  (68 , 131 | 70 ± 10  (50 , 91) | LV WT, apical septal segment (mm) † | 6.5 ± 1.1  (4.3 , 8.7) | 7.3 ± 1.2  (4.9 , 9.7) | 6.0 ± 1.0  (4.0 , 8.0) |
| LVM /h^2.7^ including PM (g/m^2.7^) † | 24 ± 4  (16 , 32) | 27 ± 4  (19 , 35) | 21 ± 3  (15 , 27) | LV WT, apical inferior segment (mm) † | 6.6 ± 1.1  (4.4 , 8.8) | 7.4 ± 1.2  (5.0 , 9.9) | 5.7 ± 1.1  (3.5 , 7.9) |
| LV RWM including PM (g/mL) † | .79 ± .16  (.47 , 1.11) | .85 ± .17  (.51 , 1.19) | .72 ± .12  (.48 , .96) | LV WT, apical lateral segment (mm) † | 6.4 ± 1.2  (4.0 , 8.8) | 7.0 ± 1.3  (4.4 , 9.7) | 5.7 ± 1.0  (3.7 , 7.8) |
| PM mass /LVM including PM (%) † | 3.7 ± 0.9  (1.9, 5.5) | 3.5 ± 1  (1.5 , 5.5) | 4 ± 1  (2.0 , 5.9) | LV anterior WT, AB ratio | .80 ± .08  (.64 , .96) | .78 ± .09  (.60 , .96) | .81 ± .08  (.65 , .97) |
| LV max/min WT ratio | 1.64 ± .30  (1.05 , 2.23) | 1.66 ± .31  (1.04 , 2.28) | 1.62 ± .30  (1.02 , 2.21) | LV septal WT, AB ratio * | .78 ± .09  (.60 , .96) | .78 ± .09  (.60 , .96) | .78 ± .09  (.60 , 95) |
| LV max WT/EDV (mm/mL) | .05 ± .01  (.03 , .07) | .05 ± .01  (.03 , .07) | .05 ± .01  (.03 , .07) | LV inferior WT, AB ratio | .80 ± .08  (.64 , .96) | .80 ± .08  (.64 , .96) | .79 ± .08  (.63 , .95) |
|  |  |  |  | LV lateral WT, AB ratio * | .80 ± .09  (.62 , .97) | .79 ± .09  (.61 , .97) | .81 ± .09  (.63 , .99) |

LV, left ventricle; EDV, end-diastolic volume; h, height; ESV, end-systolic volume; LVM: left ventricular mass; PM, papillary muscle; BSA, body surface area; RWM, relative wall mass; max, maximum; min, minimum,; WT, wall thickness; AB, apical/basal; max, maximum; min, minimum.

* Significant differences (p<0.05) among age groups on multivariable analysis

† Significant differences (p<0.05) between genders on multivariable analysis

‡ Significant differences (p<0.05) among sport categories (medium and high intensity) on multivariable analysis

**Table S4. Effect size of sport type (medium and high intensity), gender and age on LV parameters, LV wall thickness, LV and LV/RV ratios on multivariate analysis**

|  | **Sport intensity** | | | **Gender** | | | **Age** | | |
| --- | --- | --- | --- | --- | --- | --- | --- | --- | --- |
|  | **ß** | **p** | **95% CI** | **ß** | **p** | **95% CI** | **ß** | **p** | **95% CI** |
| LV EDV (mL) | .176 | <.05 | 2.56 , 29.5 | .542 | <.001 | 39.1 , 68.4 | -.009 | NS | -9.36 , 7.54 |
| LV EDV/BSA (mL/m2) | .032 | NS | -4.86 , 7.10 | .393 | <.001 | 8.78 , 21.6 | .021 | NS | -3.27 , 4.20 |
| LV EDV/h (mL/m) | .148 | NS | -.278 , 12.7 | .493 | <.001 | 15.5 , 29.6 | .066 | NS | -2.33 , 5.78 |
| LV EDV/ h^2.7^ (g/m^2.7^) | .091 | NS | -.776 , 2.53 | .346 | <.001 | 1.86 , 5.43 | .132 | NS | -.223 , 1.82 |
| LV ESV (mL) | .190 | <.05 | 1.30 , 15.6 | .408 | <.001 | 12.1 , 27.6 | -.134 | <.05 | -8.19 , -.098 |
| LV ESV/BSA (mL/m^2^) | .069 | NS | -2.08 , 4.80 | .280 | <.01 | 2.34 , 9.71 | -.186 | <.05 | -4.42 , -.185 |
| LV ESV/h (mL/m) | .177 | <0.05 | .213 , 7.55 | .345 | <.001 | 4.25 , 12.2 | -.131 | NS | -4.06 , .473 |
| LV ESV/ h^2.7^ (g/m^2.7^) | .119 | NS | -.309 , 1.60 | .251 | <.01 | .448 , 2.52 | -.131 | NS | -1.04 , .148 |
| LV EF (%) | -.092 | NS | -3.14 , .958 | .001 | NS | -2.20 , 2.23 | .334 | <.001 | 1.21 , 3.72 |
| LVM excluding PM (g) | .093 | NS | -2.50 , 17.7 | .736 | <.001 | 55.1 , 76.9 | -.024 | NS | -7.56 , 5.10 |
| LVM excluding PM /BSA (g/m^2^) | -.042 | NS | -6.27 , 3.34 | .648 | <.001 | 19.1 , 29.4 | -.038 | NS | -3.80 , 2.18 |
| LVM excluding PM /h (g/m) | .064 | NS | -2.49 , 7.57 | .721 | <.001 | 25.8 , 36.6 | .013 | NS | -2.82 , 3.46 |
| LVM excluding PM /h^2.7^ (g/m^2.7^) | .076 | NS | -.391 , 1.29 | .620 | <.001 | 4.95 , 7.89 | .078 | NS | -.389 , 1.31 |
| LV RWM excluding PM (g/mL) | -.074 | NS | -.086 , .034 | .345 | <.001 | .066 , .195 | .088 | NS | -.018 , .056 |
| PM mass (g) | .170 | NS | -.004 , 1.18 | .403 | <.001 | .905 , 2.20 | .126 | NS | -.086 , .639 |
| PM mass/BSA (g/m^2^) | .063 | NS | -.200 , .410 | .248 | <.01 | .124 , .787 | .126 | NS | -.056 , .319 |
| PM mass/LVM excluding PM (%) | .016 | NS | -.003 , .004 | -.239 | <.05 | -.010 , -.001 | .174 | NS | -.001 , .005 |
| LV AVPD, septal (%) | -.063 | NS | -1.86 , .937 | -.170 | NS | -2.85 , .124 | .087 | NS | -.453 , 1.26 |
| LV AVPD, lateral (%) | -.085 | NS | -1.18 , .681 | -.108 | NS | -2.12 , .551 | .228 | <.05 | .187 , 1.71 |
| LV sphericity index, ES | -.025 | NS | -.145 , .112 | .183 | NS | -.001 , .273 | -.013 | NS | -.085 , .074 |
| LV sphericity index, ED | -.091 | NS | -.113 , .038 | .090 | NS | -.043 , .125 | -.277 | <.01 | -.118 , -.025 |
| LVM including PM (g) | .114 | NS | -1.59 , 20.6 | .687 | <.001 | 51.1 , 75.6 | -.008 | NS | -7.36 , 6.47 |
| LVM/BSA including (g/m^2^) | -.015 | NS | -5.87 , 4.85 | .601 | <.001 | 17.2 , 28.7 | -.027 | NS | -3.87 , 2.70 |
| LVM/h including PM (g/m) | .095 | NS | -1.72 , 9.37 | .689 | <.001 | 24.5 , 36.5 | .021 | NS | -2.91 , 3.98 |
| LVM/h^2.7^ including PM (g/m^2.7^) | .015 | NS | -1.27 , 1.57 | .616 | <.001 | 5.06 , 8.10 | .057 | NS | -.525 , 1.22 |
| LV RWM including PM (g/mL) | -.006 | NS | -.060 , .056 | .350 | <.001 | .066 , .190 | -.009 | NS | -.038 , .034 |
| PM mass/LVM including PM (%) | .021 | NS | -.003 , .004 | -.215 | <.05 | -.009 , -.001 | .186 | NS | -.005 , .005 |
| PM, anterosuperior (g) | .122 | NS | -.103 , .615 | .414 | <.001 | .568 , 1.35 | .127 | NS | -.055 , .390 |
| PM, anterosuperior/BSA (g/m^2^) | -.011 | NS | -.201 , .179 | -.256 | <.01 | .085 , .495 | .119 | NS | -.040 , .193 |
| PM, posteroinferior (g) | .196 | NS | -.039, .677 | .315 | <.001 | .284 , .989 | .089 | NS | -.096 , .300 |
| PM, posteroinferior/BSA (g/m^2^) | .086 | NS | -.088 , .239 | .193 | <.05 | .064 , .371 | .075 | NS | -.061 , .143 |
| LV Max WT (mm) | - .004 | NS | -.539, .514 | .629 | <.001 | 1.92 , 3.05 | .056 | NS | -.198 , .447 |
| WTDI (mm) | .034 | NS | -.109 , .161 | .378 | <.001 | .176 , .466 | .081 | NS | -.044 , .121 |
| WT, basal anterior segment (mm) | .031 | NS | -.404 , .615 | .591 | <.001 | 1.62 , 2.71 | .040 | NS | -.231 , .396 |
| WT, basal anteroseptal segment (mm) | -.011 | NS | -.626 , .541 | .589 | <.001 | 1.88 , 3.13 | .149 | <0.05 | .006 , .718 |
| WT, basal inferoseptal segment (mm) | .073 | NS | -.229 , .654 | .584 | <.001 | 1.38 , 2.34 | .111 | NS | -.070 , .474 |
| WT, basal inferior segment (mm) | .048 | NS | -.312 , .610 | .608 | <.001 | 1.56 , 2.55 | .082 | NS | -.125 , .443 |
| WT, basal inferolateral segment (mm) | -.070 | NS | -.810 , .305 | .569 | <.001 | 1.65 , 2.85 | .080 | NS | -1.64 , .524 |
| WT, basal anterolateral segment (mm) | .009 | NS | -.503 , .514 | .526 | <.001 | 1.32 , 2.41 | .149 | NS | -.012 , .613 |
| WT, mid anterior segment (mm) | -.130 | NS | -.787 , .070 | .562 | <.001 | 1.23 , 2.16 | .072 | NS | -.142 , .389 |
| WT, mid anteroseptal segment (mm) | -.009 | NS | -.556 , .497 | .550 | <.001 | 1.45 , 2.58 | -.0.34 | NS | -.394 , .252 |
| WT, mid inferoseptal segment (mm) | .004 | NS | -.512 , .537 | .576 | <.001 | 1.59 , 2.72 | -.066 | NS | -.463 , .180 |
| WT, mid inferior segment (mm) | -.009 | NS | -.473 , .421 | .594 | <.001 | 1.46 , 2.42 | -.004 | NS | -.285 , .271 |
| WT, mid inferolateral segment (mm) | .017 | NS | -.453 , .564 | .526 | <.001 | 1.29 , 2.39 | .005 | NS | -.306 , .327 |
| WT, mid anterolateral segment (mm) | .032 | NS | -.397 , .607 | .560 | <.001 | 1.45 , 2.52 | .062 | NS | -.184 , .433 |
| WT, apical anterior segment (mm) | -.076 | NS | -.671 , .253 | .444 | <.001 | .844 , 1.85 | .035 | NS | -.227 , .346 |
| WT, apical septal segment (mm) | -.041 | NS | -.585 , .350 | .495 | <.001 | 1.05 , 2.06 | -.058 | NS | -.389 , .184 |
| WT, apical inferior segment (mm) | -.041 | NS | -.627 , .375 | .500 | <.001 | 1.15 , 2.23 | .055 | NS | -.203 , .412 |
| WT, apical lateral segment (mm) | -.034 | NS | -.555 , .363 | .517 | <.001 | 1.12 , 2.11 | -.040 | NS | -.352 , .210 |
| LV anterior WT, AB ratio | -.155 | NS | -.101 , .008 | -.162 | NS | -.113, .007 | .017 | NS | -.031 , .037 |
| LV septal WT, AB ratio | -.083 | NS | -.069 , .026 | -.115 | NS | -.085 , .019 | -.222 | <.01 | -.065 , -.007 |
| LV inferior WT, AB ratio | -.129 | NS | -.086 , .015 | .033 | NS | -.047 , .067 | -.052 | NS | -.041 , .023 |
| LV lateral WT, AB ratio | -.016 | NS | -.047 , .039 | -.004 | NS | -.049 , .047 | -.231 | <.01 | -.060 , -.007 |
| LV max WT/EDV (mm/mL) | -.134 | NS | -.007 , .001 | -.021 | NS | -0.005 , .004 | .066 | NS | -.002 , .003 |
| LV max/min WT ratio | .087 | NS | -.060 , .166 | .114 | NS | -0.05 , .199 | -.008 | NS | -.073 , .067 |
| LV / RV EDV ratio | .079 | NS | -.023 , .059 | .054 | NS | -.031 , .058 | .172 | NS | -.001 , .050 |
| LV / RV ESV ratio | .128 | NS | -.019 , .116 | .181 | NS | -.002 , .138 | .050 | NS | -.030 , .054 |
| LV / RV EF ratio | -.095 | NS | -.047 , .015 | -.149 | NS | -.061 , .006 | .013 | NS | -.018 , .021 |

LV, left ventricle; EDV, end-diastolic volume; BSA, body surface area; h, height; ESV, end-systolic volume; EF, ejection fraction; LVM: left ventricular mass; PM, papillary muscle; RWM, relative wall mass; AVPD, atrioventricular plane displacement; ES, end-systole; ED, end-diastole; WT, wall thickness; WTDI, wall thickness dispersion index; AB, apical/basal; max, maximum; min, minimum; RV, right ventricle.

**Table S5. Additional RV dimensions reference parameters summary data (mean, 95% confidence interval) for athletes of medium and high intensity sports, with gender cut-offs when applicable**

|  | **All** | **By gender** | |
| --- | --- | --- | --- |
|  |  | **Males** | **Females** |
| RVM/h (g/m) † | 37 ± 7  (23 , 50) | 42 ± 8  (26 , 58) | 32 ± 5  (22 , 42) |
| RVM/h^2.7^ (g/m^2.7^) † | 10 ± 2  (6 , 14) | 11 ± 2  (7 , 15) | 9 ± 1  (7 , 11) |
| RV EDV/h (mL/m) † | 106 ± 18  (70 , 142) | 117 ± 19  (80 , 154) | 96 ± 14  (68 , 124) |
| RV EDV /h^2.7^ (mL/m^2.7^) † | 30 ± 5  (21 , 39) | 31 ± 5  (22 , 40) | 28 ± 4  (20 , 36) |
| RV ESV/h (mL/m) * † | 42 ± 10  (22 , 61) | 45 ± 11  (23 , 66) | 38 ± 9  (21 , 55) |
| RV ESV / h^2.7^ (mL/m^2.7^) * | 12 ± 3  (6 , 17) | 12 ± 3  (6 , 17) | 12 ± 3  (6 , 17) |
| RV RWM (g/mL) † | 0.35 ± .07  (.21 , .48) | 0.37 ± .08  (.21 , .52) | 0.33 ± .06  (.22 , .45) |

RV, right ventricle; RVM, right ventricular mass; h, height; EDV, end-diastolic volume; ESV, end-systolic volume; RWM, relative wall mass.

* Significant differences (p<0.05) among age groups on multivariable analysis

† Significant differences (p<0.05) between genders on multivariable analysis

‡ Significant differences (p<0.05) among sport categories (medium and high intensity) on multivariable analysis

**Table S6. Effect size of sport type (medium and high intensity), gender and age on RV parameters on multivariate analysis**

|  | **Sport** | | | **Gender** | | | **Age** | | |
| --- | --- | --- | --- | --- | --- | --- | --- | --- | --- |
|  | **ß** | **p** | **95% CI** | **ß** | **p** | **95% CI** | **ß** | **p** | **95% CI** |
| RVM (g) | .165 | <.05 | .802 , 11.13 | .601 | <.001 | 17.6 , 29.1 | .008 | NS | -3.00 , 3.38 |
| RVM/BSA (g/m^2^) | .043 | NS | -1.95 , 3.33 | .468 | <.001 | 5.27 , 10.9 | .001 | NS | -1.62 , 1.64 |
| RVM/h (g/m) | .138 | NS | -.217 , 5.15 | .553 | <.001 | 7.81 , 13.6 | .049 | NS | -1.12 , 2.22 |
| RVM/h^2.7^ (g/m^2.7^) | .098 | NS | -.306 , 1.18 | .439 | <.001 | 1.33 , 2.92 | .100 | NS | -.180 , .739 |
| RV WT (mm) | -.080 | NS | -.042 , .019 | .374 | <.001 | .025 , .087 | -.067 | NS | -.025 , .013 |
| RV EDV (mL) | .131 | NS | -1.89 , 14.0 | .526 | <.001 | 36.1 , 65.4 | -.059 | NS | -11.6 , 5.12 |
| RV EDV/BSA (mL/m^2^) | -.059 | NS | -7.54 , 4.20 | .339 | <.001 | 6.78 , 20.4 | -.096 | NS | -6.15 , 1.69 |
| RV EDV/h (mL/m) | .095 | NS | -2.61 , 10.4 | .486 | <.001 | 14.5 , 28.5 | -.030 | NS | -4.80 , 3.30 |
| RV EDV /h^2.7^ (mL/m^2.7^) | .019 | NS | -1.48 , 1.84 | .297 | <.001 | 1.28 , 4.82 | .029 | NS | -.853 , 1.21 |
| RV ESV (mL) | .109 | NS | -2.57 , 12.2 | .362 | <.001 | 9.50 , 25.4 | -.203 | <.05 | -10.2 , -1.03 |
| RV ESV/BSA (mL/m^2^) | -.034 | NS | -4.42 , 3.01 | .168 | <.05 | .200 , 7.81 | -.237 | <.01 | -5.40 , -.830 |
| RV ESV/h (mL/m) | .074 | NS | -2.20 , 5.49 | .273 | <.001 | 2.46 , 10.7 | -.198 | <.05 | -5.11 , -.373 |
| RV ESV / h^2.7^ (mL/m^2.7^) | .013 | NS | -.944 , 1.08 | .118 | NS | -.363 , 1.80 | -.177 | <.05 | -1.24 , -.002 |
| RV EF (%) | -.003 | NS | -2.28 , 2.22 | .121 | NS | -.729 , 4.09 | .274 | <.01 | .802 , 3.58 |
| RV AVPD, lateral (%) | .016 | NS | -1.70 , 2.02 | .113 | NS | -.774 , 3.20 | .179 | NS | -.040 , 2.22 |
| RV sphericity index, ES | -.119 | NS | -.275 , .061 | -.077 | NS | -.325 , .136 | -.094 | NS | -.157 , .052 |
| RV sphericity index, ED | -.119 | NS | -.275 , .061 | -.028 | NS | -.215 , .160 | -.094 | NS | -.157 , .052 |
| RV RWM (g/mL) | .088 | NS | -.014 , .039 | .236 | <.05 | .009 , .066 | .110 | NS | -.006 , .026 |

RV, right ventricle; RVM, right ventricular mass; BSA, body surface area; h, height; WT, wall thickness; EDV, end-diastolic volume; ESV, end-systolic volume; EF, ejection fraction; AVPD, atrioventricular plane displacement; ES, end-systole; ED, end-diastole; RWM, relative wall mass
